# Particles–Matrix Bond in ZnCoO:H and ZnCoAlO:H Films: Issues of Magnetism and Spin Injection

**DOI:** 10.3390/ma16103659

**Published:** 2023-05-11

**Authors:** Yu. E. Samoshkina, M. V. Rautskii, D. S. Neznakhin, E. A. Stepanova, I. S. Edelman, Hsiung Chou

**Affiliations:** 1 Kirensky Institute of Physics, Federal Research Center KSC SB RAS, 660036 Krasnoyarsk, Russia; rmv@iph.krasn.ru; 2 Institute of Natural Sciences and Mathematics, Ural Federal University, 620002 Yekaterinburg, Russiaelena.stepanova@urfu.ru (E.A.S.); 3 Department of Physics, National Sun Yat-sen University, Kaohsiung 80424, China; northpolebearchou@g-mail.nsysu.edu.tw; 4 Department of Applied Physics, National University of Kaohsiung, Kaohsiung 81148, China

**Keywords:** thin films, zinc oxide, metallic Co nanoparticles, magnetic properties, magnetic circular dichroism spectroscopy, giant magnetoresistance effect

## Abstract

ZnCoO:H and ZnCoAlO:H films were synthesized by radio frequency magnetron sputtering in a (1 − x)Ar + xH_2_ mixed atmosphere with x = 0.2–0.5. The films contain different amounts of metallic Co particles (from 7.6% and higher) ~4–7 nm in size. The magnetic and magneto-optical (MO) behavior of the films was analyzed in combination with their structural data. The samples exhibit high values of magnetization (up to 377 emu/cm^3^) and MO response at room temperature. Two situations are considered: (1) the film magnetism is associated only with isolated metal particles and (2) magnetism is present both in the oxide matrix and in metal inclusions. It has been established that the formation mechanism of the magnetic structure of ZnO:Co^2+^ is due to the spin-polarized conduction electrons of metal particles and zinc vacancies. It was also found that in the presence of two magnetic components in the films, these components are exchange-coupled. In this case, the exchange coupling generates a high spin polarization of the films. The spin-dependent transport properties of the samples have been studied. A high value of the negative magnetoresistance of the films at room temperature (~4%) was found. This behavior was explained in terms of the giant magnetoresistance model. Thus, the ZnCoO:H and ZnCoAlO:H films with high spin polarization can be considered as sources of spin injection.

## 1. Introduction

The search and study of new magnetic-semiconductor systems that exhibit spin polarization of carriers at high temperatures is an urgent scientific and technical task. In this direction, materials based on ZnO are considered as potential candidates for semiconductor spintronics [1,2]. Zinc oxide is characterized as a wide-gap (~3.3 eV) semiconductor in which free carriers coexist with optical transparency [3,4]. At the same time, ZnO diluted with metal ions R (ZnRO) can be characterized by a high magnetic moment at room temperature [5,6,7]. However, the nature of the magnetism in such materials is still under debate. In particular, the entire magnetism of a ZnCoO sample containing metallic cobalt was usually attributed to this secondary phase [8,9,10]. Only one study of ZnCoAlO compound was found that considered the presence of magnetism inside both the oxide and the in metallic inclusions, as well as their exchange coupling through aluminum [11]. Along with the fundamental interest in the nature of magnetism in such complex systems, the materials based on ZnO are also considered as sources of spin-polarized carriers for injection into other semiconductors [12,13,14]. This makes them attractive objects for magneto-transport studies.

In recent years, the hydrogenation of ZnCoO films has become increasingly popular. It has been established that the preparation or post-synthesis treatment of the films in a hydrogen atmosphere induces or enhances the ferromagnetism of the samples [7,15,16,17,18,19,20]. Hydrogen injection into ZnCoO was usually carried out by post-irradiation with a hydrogen ion beam [7], post-synthesis plasma treatment with Ar-H_2_ mixed gas [19,20], post-synthesis annealing in H_2_ or Ar/H_2_ atmosphere [16,17], and radio frequency (RF) magnetron sputtering in Ar-H_2_ mixed gas [15,18]. At the same time, it has been shown that the temperature during the hydrogenation of samples plays a key role in the formation of their physical properties [21,22]. It is also known to hydrogenate ZnCoAlO films by post-synthesis treatment and study their magnetic properties [20]. However, data on the hydrogenation of ZnCoAlO films upon RF magnetron sputtering have been found.

In the present work, ZnCoO and ZnCoAlO films synthesized by RF magnetron sputtering in a (1 − x)Ar + xH_2_ mixed atmosphere with x = 0.2–0.5 are studied. The films contain different amounts of metallic Co particles. Then, the matrix is ZnO:Co^2+^ since part of the atomic fraction of Co^2+^ ions is distributed over the sites of the wurtzite lattice upon substitution. The magnetization behavior of these films is studied together with their structure, magneto-optical response, and magneto-transport properties. A comprehensive study has shown that the magnetism of a matrix can be due to the spin-polarized conduction electrons of the metal particles. In our opinion, such a mechanism has not been considered before. Moreover, the dominant role of the bond between particles and matrix in the physics of samples has been established.

## 2. Material and Methods

The studied films were grown on glass substrate by the standard radio frequency (RF) magnetron sputtering system using two inches Zn_0.95_Co_0.05_O and Zn_0.93_Co_0.05_Al_0.02_O targets. These targets were prepared from high purity ZnO (99.999%), Co_3_O_4_ (99.9985%), and Al_2_O_3_ (99.997%) powders by a standard two-step, solid-state reaction method. The targets synthesis is described in detail in [23,24]. The film’s deposition was carried out at a total pressure of 30 mTorr and a forward RF power of 80 W. The substrate temperature was 450 °C. Deposition time was 20 min. Four compositions of mixed gas in the sputtering chamber were used, namely Ar + (20%, 30%, 40%, and 50%) H_2_. 

The study of the morphology and chemical composition of the polycrystalline films (ex situ) showed that the thickness of the samples and Zn/Co ratio in them decrease with an increase in the hydrogen concentration in the sputtering chamber [24]. The thickness of the ZnCoO:H (CZO:H) and ZnCoAlO:H (CAZO:H) films, as well as the percentage of doping elements in them, calculated from the data of X-ray fluorescence spectrometer S4 Pioneer (Bruker, Karlsruhe, Germany) [24] are presented in Table 1. This behavior is explained by a decrease in the Zn amount in the films under the indicated deposition conditions.

Magnetic measurements were performed using a Quantum Design MPMS-XL7 EC SQUID magnetometer (Quantum Design, San Diego, CA, USA) in the temperature range 5–300 K and magnetic fields H up to 2 T directed along the film plane. The transport properties were studied on an original facility based on a KEITHLEY-2400 precision current-voltage meter in the ranges of 80 K < T < 300 K and −0.95 T < H < 0.95 T. Resistivity was measured in DC mode using a standard four-probe technique. The magnetic field was applied normally and along the film plane. Electrical contacts were formed using a two-component silver epoxy. The distance between the contacts was 1.5 mm.

A qualitative analysis was carried out of the magnetic circular dichroism (MCD) spectra measured on an original setup with a modulator in the form of a fused quartz prism with a glued piezoceramic element. The experimental technique is described in more detail in Ref. [25]. The MCD effect was measured in the normal geometry: an external magnetic field vector and the light beam were directed normal to the films plane. The modulation of the polarization state of a light wave from right-to-left circular polarization with respect to the direction of the magnetic field was used. The MCD value was measured as the difference between the optical densities of the film for right- and left-hand polarized waves divided by the sample thickness. Measurements were carried out in the spectral range 1.2–4.5 eV in a magnetic field up to 1.3 mT at the temperature 300 K. The measurement accuracy was about 10^−4^, and the spectral resolution was 20–50 cm^−1^ depending on the wavelength.

## 3. Results

### 3.1. Magnetization

**Figure 1:**
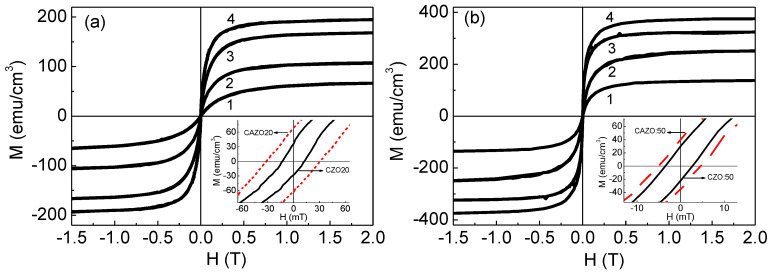
Room temperature magnetization of the CZO:H (**a**) and CAZO:H (**b**) films. The samples with an increase in the hydrogen component of 20–50% are indicated by curves 1–4, respectively. Insert (**a**) shows the hysteresis loops for the CZO:20 and CAZO:20 films at T = 20 K. Insert (**b**) shows the hysteresis loops for the CZO:50 and CAZO:50 films at T = 100 K. The contribution from the substrate was subtracted from the data.

The field dependences of the films magnetization at room temperature are presented in Figure 1. It can be seen that the CZO:H and CAZO:H samples demonstrate almost hysteresis-free behavior (the coercivity is 1–1.6 mT) with very low remanence. The magnetization value increases from sample to sample with an increase in Co content in each series. The saturation magnetization (M_S_) value of the CAZO:20–CAZO:50 films at 2 T is almost twice that of the CZO:20–CZO:50 films (Table 2). It should be noted that the shape of the M-H curves for the studied films is typical of both superparamagnetic particles and dilute magnetic semiconductors [26,27]. Thermomagnetic magnetization curves allow one to choose between these two situations [28]. Such curves were recorded during heating of the samples after their cooling in a magnetic field (FC) or in the absence of a magnetic field (ZFC). 

**Figure 2:**
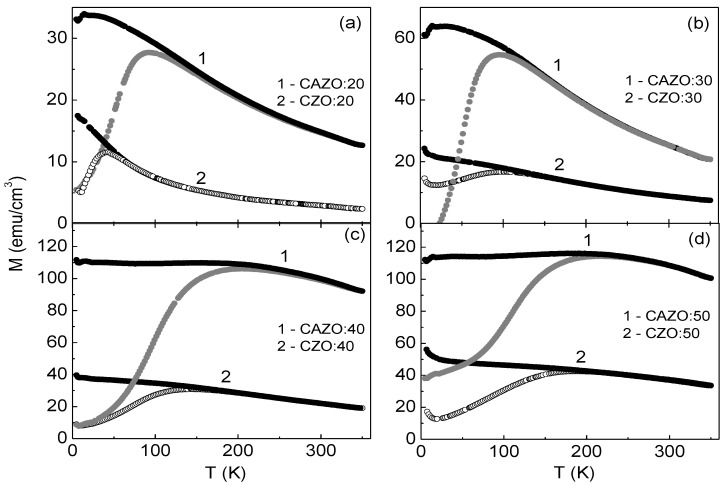
FC-ZFC temperature dependences of the magnetization for the CZO:H and CAZO:H films at H = 10 mT.

The FC and ZFC temperature dependences of the magnetization for all studied samples shown in Figure 2 exhibit a divergence typical for an ensemble of superparamagnetic nanoparticles with a blocking temperature (T_b_) determined by the maximum on the ZFC curves. For the CZO:20-CZO:50 films, T_b_ increases from 40 to 184 K, respectively (Table 2). In the case of the CAZO:20-CAZO:50 samples, T_b_ is initially higher and increases from 90 to 217 K, respectively (Table 2). At temperatures below T_b_, magnetic particles are characterized by M-H magnetization curves with non-zero coercive field (for example, see inserts in Figure 1a,b). The H_C_ value at T = 20 K is 12 and 31 mT, whereas the M_r_ value is 36 and 69 emu/cm^3^, for the CZO:20 and CAZO:20 samples, respectively. For the CZO:50 and CAZO:50 films, the H_C_ value at T = 100 K is 3 and 5 mT and the M_r_ is 30 and 39 emu/cm^3^, respectively. 

Park et al. noted that the solubility limit of Co in ZnO is approximately 12% [8]. Above 12%, metallic cobalt clusters were found in Zn_1−x_Co_x_O films. Taking into account the relatively high content of Co in the studied films (Table 1), one can suppose the nanoparticles described by the shown FC-ZFC curves to consist of metallic Co. Though the X-ray diffraction patterns obtained for the films exhibited diffraction peaks corresponding only to the ZnO crystal [23,24], the element-selective XANES spectroscopy at the Co K-edge confirmed the presence of metallic cobalt in the CZO:20 and CZO:30 films. In combination with XRFA data, the relative content of metallic cobalt in the films was estimated as 7.6% and 13%, respectively [29]. The rest of the samples were not examined with XANES. However, it is reasonable to assume that superparamagnetic particles in them also consist of metallic Co. Magneto-optical spectra of the films that will be analyzed below can also be considered as indirect evidence of the presence of metallic Co nanoparticles in them.

The average size of the included Co particles (<D>) was estimated by the Bean–Livingston formula T_b_ = K_eff_ (4πr^3^/3)/25 k_B_ [30], where K_eff_ is the energy of the effective magneto-crystalline anisotropy (4.3 × 10^5^ J/m^3^ [29,31]), k_B_ is Boltzmann’s constant, and r is the particles radius. The <D> values for the studied samples are shown in the Table 2. These values do not differ much for the CZO:H and CAZO:H series and vary from ~4 to ~7 nm.

It should be noted that the ratio of the M_S_ values of the CZO:20 and CZO:30 samples at room temperature (~67 and ~107 emu/cm^3^) agrees well with the ratio of the relative content of metallic Co in the samples (7.6% and 13%, respectively). This suggests that the magnetism of these films is mainly due to the Co particles. Based on the data on the magnetic saturation of the films, all their magnetism can be attributed only to the Co particles, the content of which increases with an increase in the hydrogen component. However, magneto-optics allows a broader view of the various magnetic contributions in such materials.

### 3.2. Magneto-Optic Spectroscopy

Magneto-optical MCD spectra of the studied films were measured earlier and presented in [24]. Note that the MCD effect is observed in transmitted light and characterizes the absorption of the medium. This method is extremely informative. It reflects not only the magnetic behavior of the sample, but also probes the spin-polarized electronic states in it. In addition, the MCD effect is observed directly at the electronic transition frequency and excludes the contribution of the nonmagnetic component (substrate/matrix). For the CZO:H and CAZO:H films, an increase in the MCD signal was found in the entire energy range with an increase in the hydrogen component. At the same time, in each pair of the samples, the MCD signal of CAZO:H is noticeably higher. It was also shown that the contributions of metallic Co particles and the ZnO:Co^2+^ matrix dominate in different regions of the MCD spectrum [29]. Moreover, it was found that with an increase in the filling density of the particles, the MCD signal increases. In turn, the particles size affects the redistribution of the intensity of the two main contributions with centers of gravity of 1.5 and 2.7 eV, which determine the wide MCD band. This behavior is explained by particle agglomeration.

Therefore, a detailed analysis of MCD spectroscopy is especially important when studying the magnetic nature of the CZO:H and CAZO:H samples. For qualitative analysis, the MCD spectrum of Co nanoparticles (Co-NPs) dispersed in a SiO_2_ matrix with <D> = 7.7 nm was additionally measured. The temperature and magnetic field were 300 K and 1.3 T, respectively. The synthesis and structure of the samples are described in Ref. [29].

**Figure 3:**
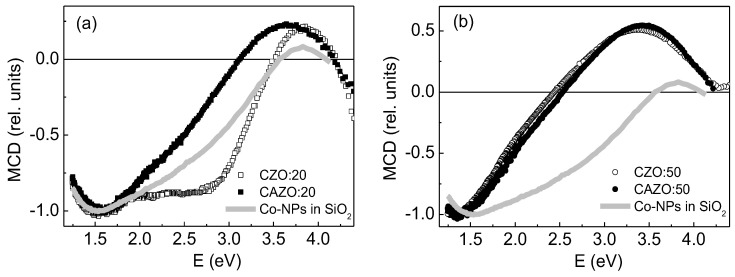
Normalized MCD spectra at H = 1.3 T and T = 300 K: (**a**)—CZO:20 and CAZO:20 films, as well as Co-NPs in SiO_2_ with <D> = 7.7 nm; (**b**)—CZO:50 and CAZO:50 films, as well as Co-NPs in SiO_2_ with <D> = 7.7 nm.

The Figure 3 shows the normalized MCD spectra of the CZO(CAZO):20 and CZO(CAZO):50 films, as well as for the Co-NPs in SiO_2_. There is a clear rearrangement between the spectra of the films with different hydrogen markings. The broad maximum observed below zero determines the contribution of metallic Co particles to the signal, whereas the broadening of the positive maximum upon going from the CZO(CAZO):20 to CZO(CAZO):50 films indicates the contribution of the magnetized ZnO:Co^2+^ matrix. Previously, for the ZnO:Co^2+^ magnetic matrix, a positive MCD signal was observed in the region of 1.5–3.3 eV [7,32,33]. 

**Figure 4:**
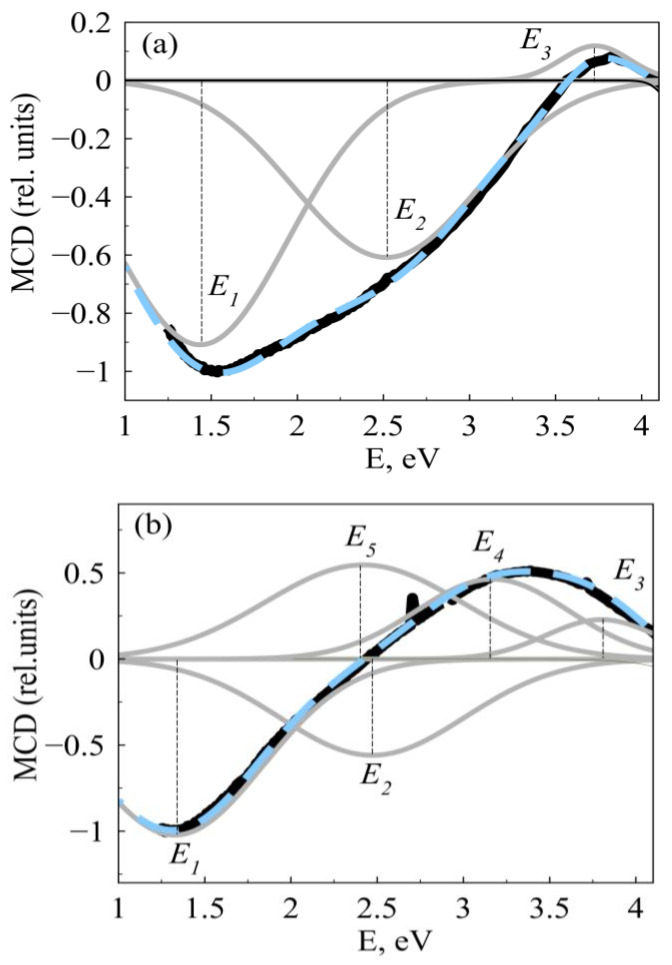
Scheme of the decomposition of the MCD spectra into main E_1_–E_5_ lines: (**a**)—Co-NPs in SiO_2_ with <D> = 7.7 nm; (**b**)—the CZO:50 film. Bold solid curve is the experimental MCD spectrum; dashed curve is the contributions sum.

To estimate the contributions to the MCD signal, the spectra of the CZO(CAZO):20 and CZO(CAZO):50 films were decomposed into components together with the MCD spectra of Co-NPs in SiO_2_. The decomposition was carried out according to the minimum number of the Gaussian lines. The Gaussian line amplitude (a), position (x_0_), and full width at half maximum (dx) were the fitting parameters. The MCD spectra of Co-NPs in SiO_2_ are well described by the E_1_–E_3_ lines at 1.4, 2.5, and 3.7 eV, respectively (scheme is shown in Figure 4a). These lines formed the basis for the MCD spectra decomposition of the CZO:H and CAZO:H films. The MCD spectra of the CZO:20 and CAZO:20 films were described by four lines. The E_1_–E_3_ lines refer to the contribution of the metallic Co particles. The E_4_ line near 3.4 eV is attributed to the contribution of the ZnO:Co^2+^ matrix. A similar line is observed in the MCD spectra of paramagnetic CZO samples [17,23,34]. The nature of this line, in our opinion, is determined by the spin polarization of electrons in the ZnO conduction band. In the case of the CZO:50 and CAZO:50 films, the E_1_–E_5_ lines at 1.4, 2.48, 3.7 eV, 3.2 eV, and 2.46 eV, respectively, are observed (Figure 4b). The broad E_4_ and E_5_ lines already indicate the contribution of the highly spin-polarized magnetized ZnO:Co^2+^ matrix.

To correctly calculate the intensity of the decomposition lines, the MCD spectra were plotted depending on the cm^−1^ value. The position of the Gaussian lines is presented in Table 3. The values of x_0_ and dx for the studied samples are in good agreement with each other. At the same time, the line amplitude for the CAZO:H films is noticeably higher than for the CZO:H films. The intensities of the identified lines are presented in Table 4.

**Figure 5:**
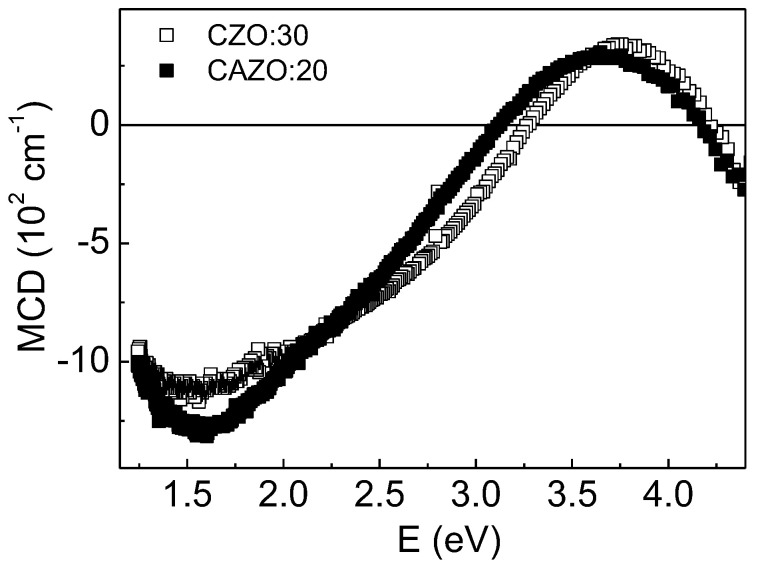
Room temperature MCD spectra of the CAZO:20 and CZO:30 films H = 1.3 T.

The contribution to the MCD from the metallic Co particles was taken as the intensity sum of the E_1_–E_3_ lines (I_Co_). The contribution from the magnetized ZnO:Co^2+^ matrix was taken as the intensity sum of the E_4_ and E_5_ lines (I_ZnO_). An increase in the I_Co_ value upon transition from CZO(CAZO):20 to CZO(CAZO):50 suggests an increase in the concentration of the metallic Co particles in the films. It was traced that the I_Co_ value for the CAZO:20 film is 1.85 times greater than the I_Co_ value for the CZO:20 sample. According to XANES data [29], the relative content of metallic cobalt in the CZO:20 film is 7.6%. Consequently, this indicator should increase by 1.85 and reach ~14% for the CAZO:20 film. This magnitude is in good agreement with the CZO:30 film data. The MCD spectrum of the CAZO:20 film is close to that of the CZO:30 film (Figure 5). According to XANES data [29], the relative content of metallic cobalt in a CZO:30 film is 13%. It is noteworthy that the values of I_Co_ and I_ZnO_ for the CAZO:50 film are 2.7 times higher than for the CZO:50 film. Such a proportional increase in contributions indicates a tight coupling between the particles and the matrix. This behavior will be discussed in more detail below.

### 3.3. Electrical Resistance Measurements

**Figure 6:**
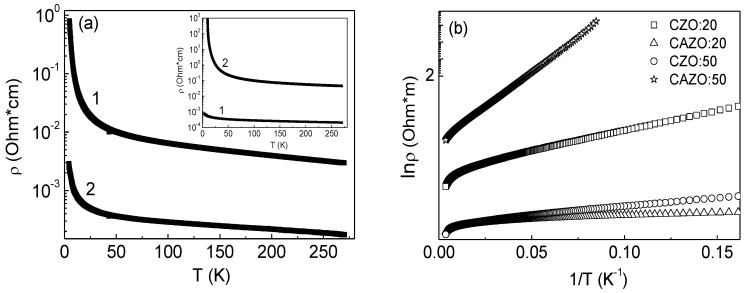
(**a**)—Temperature dependence of resistance ρ(T) in zero magnetic field represented on a logarithmic scale for the CZO:20 (curve 1) and CZO:50 (curve 2) films. The inset shows ρ(T) for the CAZO:20 (curve 1) and CAZO:50 (curve 2) films. (**b**)—Dependence of lnρ vs. T^−1^ for the specified films.

The temperature dependences of the resistivity of the CZO(CAZO):20 and CZO(CAZO):50 films indicate their semiconductor character (Figure 6a). It is noted that the sample resistivity decreases when going from CZO:20 to CZO:50, whereas the resistivity increases when going from CAZO:20 to CAZO:50 (despite the fact that the relative Al content increases). This behavior of the studied films was discussed in [24]. Two competing mechanisms that affect the resistivity, namely, the appearance of electron donors and grain-boundary scattering, were considered. Obviously, with a decrease in a film thickness, the density of defects at the grain boundaries increases. This increases the grain-boundary scattering of free electrons and leads to an increase in the sample resistivity. Apparently, the grain-boundary scattering predominates in the case of the CAZO:H films.

The linear relation of lnρ vs. (T^−n^) is best obtained at n = 1 (Figure 6b). This behavior describes the hopping mechanism of charge carrier transfer to the nearest centers and thermal delocalization of charge carriers. Thus, it should be concluded that the hopping mechanism is realized at low temperatures, whereas at higher temperatures, there is a transition to thermal delocalization of charge carriers.

**Figure 7:**
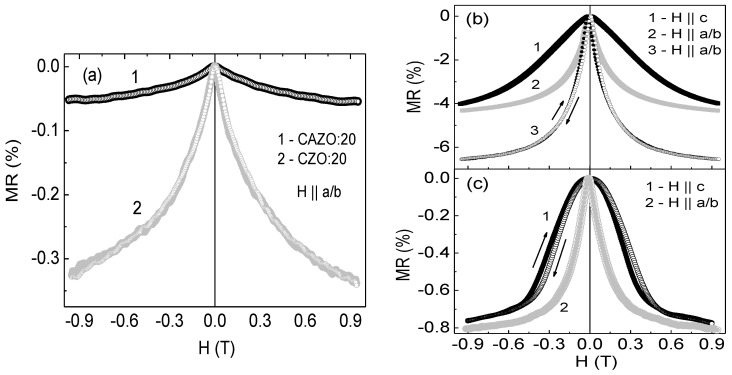
Magnetoresistance value of the CZO(CAZO):20 (**a**), CAZO:50 (**b**), and CZO:50 (**c**) films as a function of magnetic field up to 0.95 T. (**a**,**c**)—Curves 1 and 2 were measured at T = 80 K; (**b**)—Curves 1 and 2 were measured at room temperature, curve 3 corresponds to the T = 80 K.

The field dependences of the transverse (H || c) and longitudinal (H || a/b) magnetoresistance (MR) of the films defined as MR = [ρ(H) − ρ(0)]/ρ(0) are shown in the Figure 7. The CZO:20 and CAZO:20 samples exhibit a low negative MR value within 0.32% (Figure 7a). It is noteworthy that the MR value of CAZO:20 is lower than that of CZO:20 despite the doubled content of the Co metal (~14%). The CAZO:50 film has the highest negative MR value, which is ~4% at room temperature and ~6.5% at 80 K in the magnetic field of 0.95 T applied in both orientations (Figure 7b). At the same time, the MR value of the CZO:50 film does not exceed 1% at 80 K. It should be noted that the MCD spectra shape of the CAZO:50 and CZO:50 samples is practically the same (Figure 3b). These samples can only be distinguished by the larger MCD signal for CAZO:50. This regularity indicates a higher spin-polarization in the CAZO:50 film.

In a number of scientific reports, the presence of a negative MR effect in such materials is explained by spin-dependent tunneling between Co particles through the ZnO:Co^2+^ barrier [13,14,35]. At the same time, these materials should exhibit a non-linear behavior of current-voltage characteristics (CVC). In our case, the samples show a linear behavior of the CVC (not shown). Thus, the implementation of the giant MR (GMR) effect in them is most probable. The GMR effect is typical for granular Co-Ag, Mn/Co-ZnO, and Fe-In_2_O_3_ films [36,37,38].

**Figure 8:**
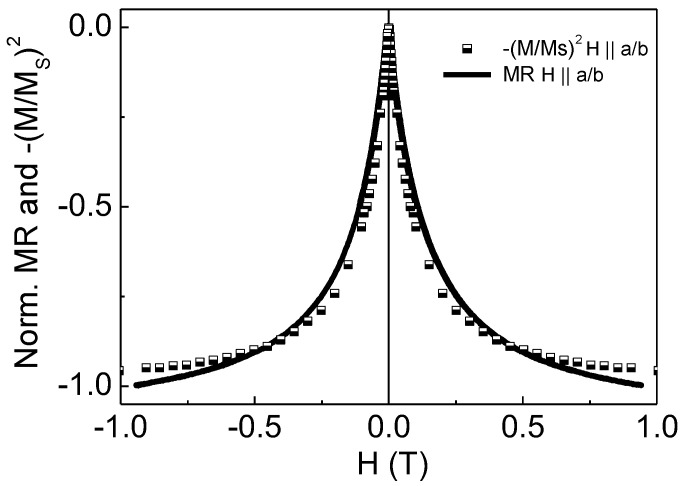
Relation between MR and −(M/M_S_)^2^ values for the CAZO:50 film at room temperature.

If the tight coupling between the matrix and the particles is responsible for the GMR effect of the CAZO:50 film, then the MR~−(M/Ms)^2^ relation must be satisfied [39]. Such a relation was considered only for room-temperature data (Figure 8). It can be seen that there is good agreement between the curves.

## 4. Discussion

The combination of the data obtained shows that the magnetization of the CZO:H and CAZO:H films, as well as their MO response increase at room temperature with an increase in the relative Co content in the samples. This behavior is explained by an increase in the amount of metallic cobalt in the films. The partial XANES data [29] and an increase in the MCD signal near 1.5 eV indicate this. At the same time, an increase in the intensity of the MCD signal of the CZO:50 and CAZO:50 films in the high-energy region indicates a magnetic contribution from the ZnO:Co^2+^ matrix.

The formation of the ZnO:Co^2+^ magnetic structure in the studied samples is a complex mechanism. The synthesis of the CZO:H and CAZO:H films under the conditions used showed that zinc is etched with hydrogen, and therefore the films thickness decreases, while the relative content of Co and Al increases (Table 1). At the same time, the number of generated zinc vacancies (V_Zn_) also increases in the samples. This behavior was characteristic of ZnO:Al films annealed in a hydrogen atmosphere [40]. An increase in the relative Co content leads to an increase in the Co-H-Co ferromagnetic units in the ZnO lattice [18,22,33,41]. In turn, an increase in the relative Al content in the CAZO:H films induces oxygen vacancies (V_O_) and generates free electrons [20,40]. However, Al donors are deactivated by V_Zn_ defects. At the same time, V_O_ defects are passivated by hydrogen [20,40]. Consequently, the number of the Co-H-Co units should be reduced. 

Based on this scheme, the ZnO:Co^2+^ magnetic moment for the CZO:50 film can be caused by the ferromagnetic Co-H-Co units and/or ferromagnetic defects-related units. The strong correlation between ferromagnetism and the number of V_Zn_ defects has been confirmed for undoped ZnO films [42]. The magnetic moment of the films was attributed to unpaired 2p electrons at the O sites surrounding the V_Zn_ defects. In the case of the CAZO:50 film, the number of the ferromagnetic Co-H-Co and the defects-related units should be reduced, which is inconsistent with the data obtained. Therefore, to explain the ZnO:Co^2+^ magnetic moment for the CZO:50 and CAZO:50, a carrier-mediated mechanism was considered [43,44].

The MCD spectra of Co-NPs in SiO_2_ [29] showed that a positive MCD signal in the region of 3.8 eV appears with an increase in the particle concentration and their average size (Figure 4a). This signal is due to spin-polarized conduction electrons and reflects, probably, the onset of the percolation process with the formation of metallic Co clusters. It was found that a similar signal is characteristic of the MCD spectra of the studied films (E_3_ line in Figure 4b). With an increase in the intensity of this signal, the intensity of E_4_ line also increases (Table 4). It is assumed that some of these electrons are captured at the V_Zn_ site and excited into the ZnO:Co^2+^ conduction band. This causes high spin polarization in the CZO:50 and CAZO:50 films. Thus, the MO response of the ZnO:Co^2+^ matrix can be explained by the Ruderman–Kittel–Kasuya–Yosida (RKKY) interaction [45]. According to the RKKY interaction, magnetic moment in the nonmagnetic ZnO:Co^2+^ matrix arises due to an indirect exchange interaction between localized Co^2+^ spins through collectivized conduction electrons.

The MCD spectroscopy of the studied samples separates the contributions from the matrix and particles. The MO data obtained indicate the presence of an exchange interaction between the magnetized ZnO:Co^2+^ matrix and metallic Co particles in the CZO:50 and CAZO:50 films. In particular, the I_Co_ ratio between the films of CZO:50 and CZO:20 films is ~5.3 (Table 4). Thus, the CZO:50 film should contain about 40% cobalt metal if the CZO:20 film contains 7.6% [29]. However, this does not agree with the overall value of the relative Co content in the film, which is 27.8% (Table 1). At the same time, the I_Co_ values for the films of CZO:20 and CAZO:20 correlate well. It has been determined above that the CAZO:20 film contains about 14% of metallic Co particles. In addition, it was observed that the I_Co_ and I_ZnO_ values for the CAZO:50 film are 2.7 times greater than for the CZO:50 film. This proportional growth of both contributions clearly indicates an exchange coupling between them. In this case, the considered exchange interaction is the largest for the CAZO:50 film.

It should be concluded that spin-polarized charges induce spin-dependent transport in samples to varying degrees. This is evidenced by the value of the GMR effect (Figure 7). The inequality GMR_CZO:20_ > GMR_CAZO:20_ can be explained by the large number of V_Zn_ defects in the CAZO:20 film. The correlation between the films thickness and the amount of zinc in the samples was traced previously [24]. The CAZO:20 film is thinner and therefore contains more zinc vacancies. Apparently, the double content of metallic Co particles in the CAZO:20 film is insufficient for the V_Zn_ passivation. It is assumed that a certain ratio between the volume fraction of particles and zinc vacancies establishes the particle-V_Zn_-matrix bond.

The high degree of the spin polarization of the CAZO:50 sample enhances the GMR effect at room temperature. The GMR value in this case is ~4%. However, tuning the particle–matrix bond through zinc vacancies is promising. Thus, CZO:H and CAZO:H films can be considered as sources of spin injection into pure semiconductors such as ZnO-Co composite films [14] or Co/ZnO and Co/ZnAlO heterostructures [13]. It should be noted that a clear Al effect on the magnetic and transport properties has not been established. However, its participation in the RKKY interaction is likely.

## 5. Conclusions

Along with the structure, the magnetic, magnetooptical, and magnetotransport properties of the ZnCoO:H and ZnCoAlO:H films with different contents of metallic Co particles ~4–7 nm in size were studied. It has been established that a magnetic order appears in the ZnO:Co^2+^ matrix depending on the number of particles and vacancies in the samples. Its mechanism is explained by the RKKY interaction and is due to the coupling of particles with the matrix through zinc vacancies. It was also found that in the presence of two magnetic components in the films, these components are exchange-coupled. This leads to high spin polarization in the samples and large values of the GMR effect (4% at room temperature and 7% at 80 K). Thus, the ZnCoO:H and ZnCoAlO:H films with high spin polarization can be considered as sources of spin injection. As a result, the dominant role of the bond between particles and matrix in the physics of samples has been established. Note that such bond can also be traced in other dilute semiconductors. The data obtained indicate the possibility of tuning this bond for practical spin-based-device applications.

## Figures and Tables

**Table 1:** The thickness of the ZnCoO:H (CZO:H) and ZnCoAlO:H (CAZO:H) films, as well as percentage of doping elements in them according to XRFA data [24].

Sample	Gas Mixture	Film Thickness (XRFA), nm	Co, % (Δ = ±0.05)	Al, % (Δ = ±0.05)
CZO:20 CAZO:20	Ar + 20% H_2_	71.7 36.9	18.9 18.2	- 7.3
CZO:30 CAZO:30	Ar + 30% H_2_	53.5 18.8	21.7 25.0	- 10.0
CZO:40 CAZO:40	Ar + 40% H_2_	37.0 18.0	25.6 25.6	- 10.2
CZO:50 CAZO:50 *	Ar + 50% H_2_	31.6 14.1	27.8 *	- *

* Zn/Co ratio was not determined for the film because of its very small thickness.

**Table 2:** Average size of the Co particles (<D>) in ZnCoO:H (CZO:H) and ZnCoAlO:H (CAZO:H) films, as well as magnetic and magneto-transport characteristics of the samples.

Sample	Gas Mixture	M_S_ at 300 K (emu/cm^3^)	M_r_ at 300 K (emu/cm^3^)	T_b_ (K)	<D> (nm)	MR at 300 K (%)
CZO:20	Ar + 20% H_2_	67	0.5	40	3.9	0.17
CAZO:20		137	0	90	5.2	0.06
CZO:30	Ar + 30% H_2_	107	1.3	99	5.3	-
CAZO:30		251	4	99	5.3	-
CZO:40	Ar + 40% H_2_	168	2	142	6	-
CAZO:40		325	7	205	6.8	-
CZO:50	Ar + 50% H_2_	195	10.5	184	6.6	0.3
CAZO:50		377	25	217	6.9	4

**Table 3:** Position (x_0_) and full width at half maximum (dx) of the Gaussian lines in the MCD spectrum at room temperature. The data are presented for the CZO:H and CAZO:H films, as well as the Co-NPs in SiO_2_ with <D> = 7.7 nm.

Sample	E_1_ (cm^−1^)		E_2_ (cm^−1^)		E_3_ (cm^−1^)		E_4_ (cm^−1^)		E_5_ (cm^−1^)	
	x_0_	dx	x_0_	dx	x_0_	dx	x_0_	dx	x_0_	dx
**Co-NPs in SiO_2_**	**11,566**	**4794**	**20,306**	**5063**	**30,094**	**2077**	-	-	-	-
CZO:20	11,648	4794	21,251	4940	31,004	2357	28,814	2856	-	-
CAZO:20	11,805	4949	20,871	5221	30,701	2377	26,723	4510	-	-
CZO:50	10,608	4800	20,000	4957	29,313	3194	24,665	4754	19,712	5630
CAZO:50	11,120	4814	20,006	5061	29,903	2930	25,793	3748	19,838	5717

**Table 4:** Intensity of the Gaussian lines in the MCD spectrum of the CZO:H and CAZO:H films, as well as the Co-NPs in SiO_2_ with <D> = 7.7 nm.

Sample	I_E1_	I_E2_	I_E3_	I_E4_	I_E5_
Co-NPs in SiO_2_	9300.39	6626.99	536.72	-	-
CZO:20	4.78 × 10^6^	4.82 × 10^6^	0.54 × 10^6^	0.47 × 10^6^	-
CAZO:20	12.12 × 10^6^	5.75 × 10^6^	0.88 × 10^6^	3.68 × 10^6^	-
CZO:50	28.84 × 10^6^	16.37 × 10^6^	4.50 × 10^6^	10.21 × 10^6^	17.26 × 10^6^
CAZO:50	81.38 × 10^6^	41.29 × 10^6^	11.18 × 10^6^	26.98 × 10^6^	47.75 × 10^6^

## Data Availability

The data presented in this study are available on request from the corresponding author after obtaining the permission of an authorized person.
